# Therapeutic Potential of Cannabidiol in Dysbiosis-Related Oral Biofilm Diseases: Antibiofilm, Antivirulence and Host Response Evidence

**DOI:** 10.3390/ph19081221

**Published:** 2026-08-03

**Authors:** Jiaqi Zhu, Xinyan Huang, Shuangyue Wu, Xiaoran Xu, Siyuan Wu, Yuankun Zhai, Jianhang Bao

**Affiliations:** 1 School of Stomatology, Henan University, Kaifeng 475001, Chinaxinyan_huang@henu.edu.cn (X.H.);; 2 Kaifeng Key Laboratory of Periodontal Tissue Engineering, Kaifeng 475000, China; 3 Eastman Institute for Oral Health, University of Rochester Medical Center, Rochester, NY 14642, USA; 4 Peking University Hospital of Stomatology Sanya Division (Sanya Stomatology Center), Sanya 572013, China

**Keywords:** cannabidiol, oral biofilms, caries, periodontal disease, dysbiosis

## Abstract

Dysbiosis-related oral biofilm diseases, particularly dental caries and periodontal diseases, pose major global health challenges because ecological shifts within oral microbial communities enhance biofilm virulence, resilience, and host inflammatory responses. Cannabidiol (CBD), a non-psychoactive phytocannabinoid with antimicrobial, antibiofilm, immunomodulatory, and antioxidant properties, has attracted increasing interest as an investigational, ecology-oriented adjunct for oral health applications. This narrative review evaluates current antibiofilm, antivirulence, and host response evidence for CBD in dysbiosis-related oral biofilm diseases, with emphasis on dental caries and periodontal diseases and selected supportive evidence from other oral biofilm-associated conditions. Current evidence suggests that CBD can inhibit biofilm formation, attenuate cariogenic and fungal virulence traits, modulate periodontal inflammation and immunity, and support tissue-protective responses. However, most evidence remains preclinical and model-dependent, particularly in caries research, and CBD’s hydrophobicity, limited stability, uncertain dose windows, and incomplete microbiome-level evidence remain major barriers to translation. Future studies should clarify CBD’s ecological effects on oral microbial communities, define clinically relevant dosing and exposure timing, and develop oral-retentive delivery systems.

## 1. Introduction

Oral biofilms, the microbial communities, adhere to tooth surfaces and mucosa, encapsulated by extracellular polymetric substances, forming highly organized structures [1,2]. In a healthy state, the oral microbiota maintains a dynamic balance with the host, contributing to oral ecological and immune system homeostasis. Dysbiosis within oral biofilms, leading to increased pathogenicity, heightens the risk of diseases such as dental caries and periodontal disease, two of the most prevalent chronic conditions worldwide [3]. Due to their complex composition, spatial organization, and physiological characteristics, biofilms exhibit significant resistance to interventions, with the extracellular matrix acting as physical barriers hindering drug penetration and immune clearance. Moreover, diverse microenvironments within biofilms harbor dormant or slow-proliferating pathogens and facilitate the transfer of resistance genes, thereby accelerating antimicrobial resistance and complicating clinical treatments [4,5,6]. Recent research has shifted the focus from single bacterial infections to microbial dysbiosis as a key etiological factor in these diseases, like dental caries and periodontal diseases [1,7,8,9]. With the limitations of current interventions [10,11], research is increasingly focused on non-antibiotic alternatives, such as probiotics and plant bioactive extracts. These approaches aim to disturb biofilm defenses, target pathogen interactions, and restore microbial balance, ultimately achieving long-term control by reducing virulence rather than eradicating microorganisms.

Cannabidiol (CBD), a non-psychoactive phytocannabinoid with multi-target pharmacological properties, including rebalancing gut microbial homeostasis, anti-inflammatory, immunoregulatory, and antioxidant effects, has garnered attention in dentistry, particularly regarding dysbiosis-related oral biofilm diseases. Moreover, the rapidly expanding global market for CBD-containing products and their increasing use by consumers underscore the need for systematic evaluation of their efficacy and safety from both translational and public health perspectives [12,13]. Despite existing reviews on CBD in dentistry [14,15], a dysbiosis-centered synthesis linking CBD’s antibiofilm, antivirulence, and host response effects in oral biofilm diseases remains lacking. This narrative review therefore synthesizes the current evidence for CBD in dysbiosis-related oral biofilm diseases, focusing on dental caries and periodontal diseases as representative conditions. We further organize the evidence around three mechanistic domains: inhibition of biofilm formation and persistence, attenuation of microbial virulence traits, and modulation of host inflammatory, immune, oxidative, and regenerative responses.

## 2. Literature Search and Critical Appraisal Methods

### 2.1. Literature Search Strategy

A structured literature search was conducted in PubMed, Web of Science Core Collection, and Google Scholar from database inception to 31 March 2026 for English-language studies using combinations of terms related to CBD, oral biofilms, dysbiosis, dental caries, periodontal disease, endodontic conditions, oral candidiasis, and representative oral microorganisms.

### 2.2. Study Selection and Eligibility

**Figure 1:**
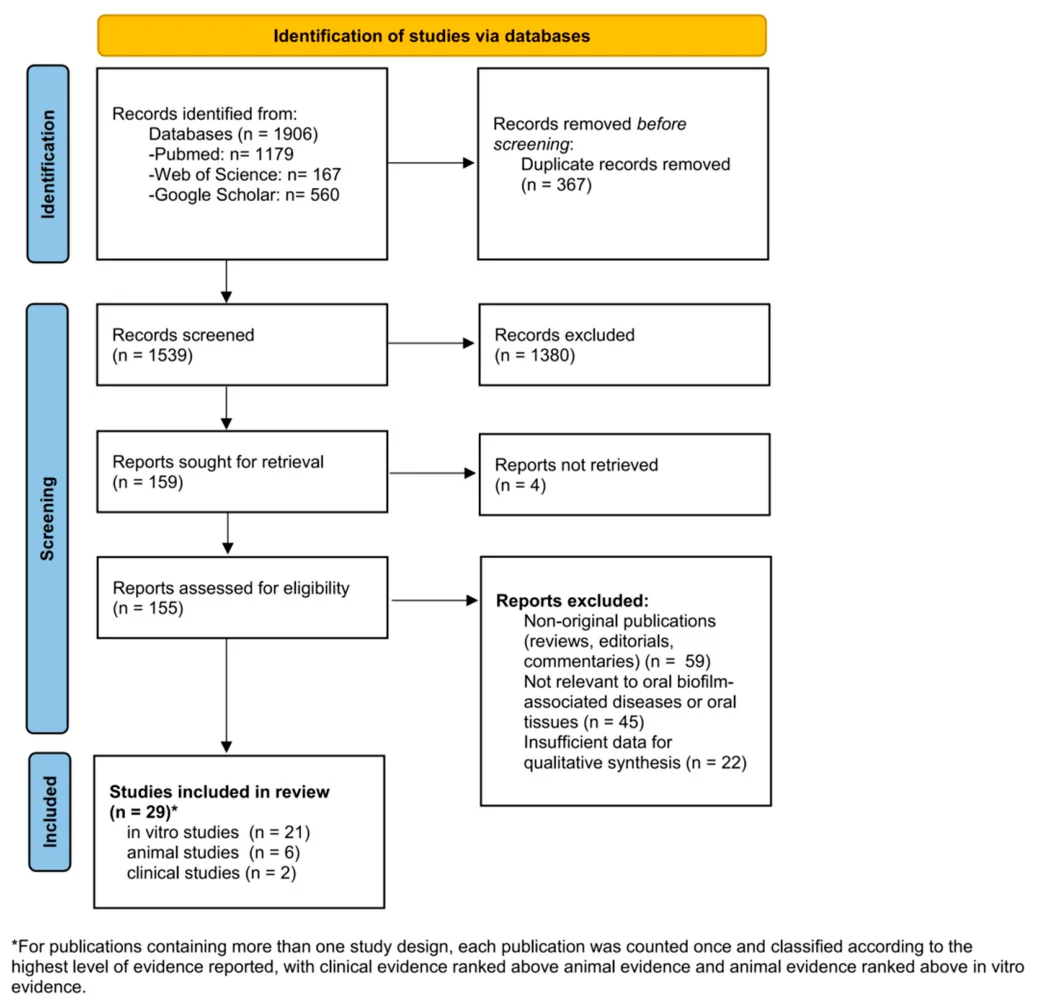
PRISMA-style flow diagram of the identification, screening, eligibility assessment, and inclusion of studies.

The search yielded 1906 records. After removing 367 duplicates, 1539 records were screened, of which 1380 were excluded. Of the 159 reports sought for retrieval, four were unavailable. The remaining 155 full-text reports were assessed for eligibility, of which 126 were excluded, leaving 29 studies included in the review. The study selection process is shown in Figure 1.

### 2.3. Critical Appraisal Approach

Given the heterogeneity of the included study designs, methodological features were assessed qualitatively according to model relevance, replication, comparator selection, CBD dose and exposure, outcome assessment, and, where applicable, randomization, blinding, and clinical study design.

## 3. Characteristics and Burden of Dysbiosis-Related Oral Biofilm Disease

### 3.1. Dental Caries

Dental caries, a typical biofilm-dependent disease, profoundly compromises quality of life [16], and affects over 2.6 billion individuals worldwide, imposing an estimated annual economic burden exceeding 700 billion dollars [3,17]. The age-standardized incidence rate of dental caries in adults is 29,777.03 per 100,000 [18], underscoring an urgent demand for effective, sustainable, and accessible management strategies.

From an ecological perspective, dental caries develops when supragingival biofilms tip from a symbiotic state into a dysbiotic, acidogenic community due to the complex interplay between the host, diet, and oral microbiota, particularly due to frequent sugar consumption [19,20]. This shift in understanding has prompted re-evaluation of the traditional emphasis on the cariogenic role of *Streptococcus mutans*, which, owing to its potent acidogenic and aciduric capacities and a remarkable ability to adhere firmly to enamel via glucosyltransferase (Gtfs)-mediated extracellular polysaccharide (EPS) synthesis, has long been considered as the principal cariogenic bacterium [21,22]. Although the average representation of *S. mutans* can be as low as 0.1% in subjects with low caries activity [23], *S. mutans* and its synergistic partner *Candida albicans*, a fungus associated with early childhood caries (ECC) and root caries, form highly pathogenic, intertwined biofilms with enhanced biomass and EPS production [24,25,26]. These organisms therefore remain highly relevant for understanding disease pathogenesis and for developing targeted management strategies, particularly in severe disease and vulnerable populations [27,28]. Imbalance of these two species within native oral microflora drives the development of a dysbiotic, cariogenic oral ecosystem [16]. Consistently, clinical studies have reported associations between the co-detection of these microorganisms and both ECC and root caries [29,30].

**Figure 2:**
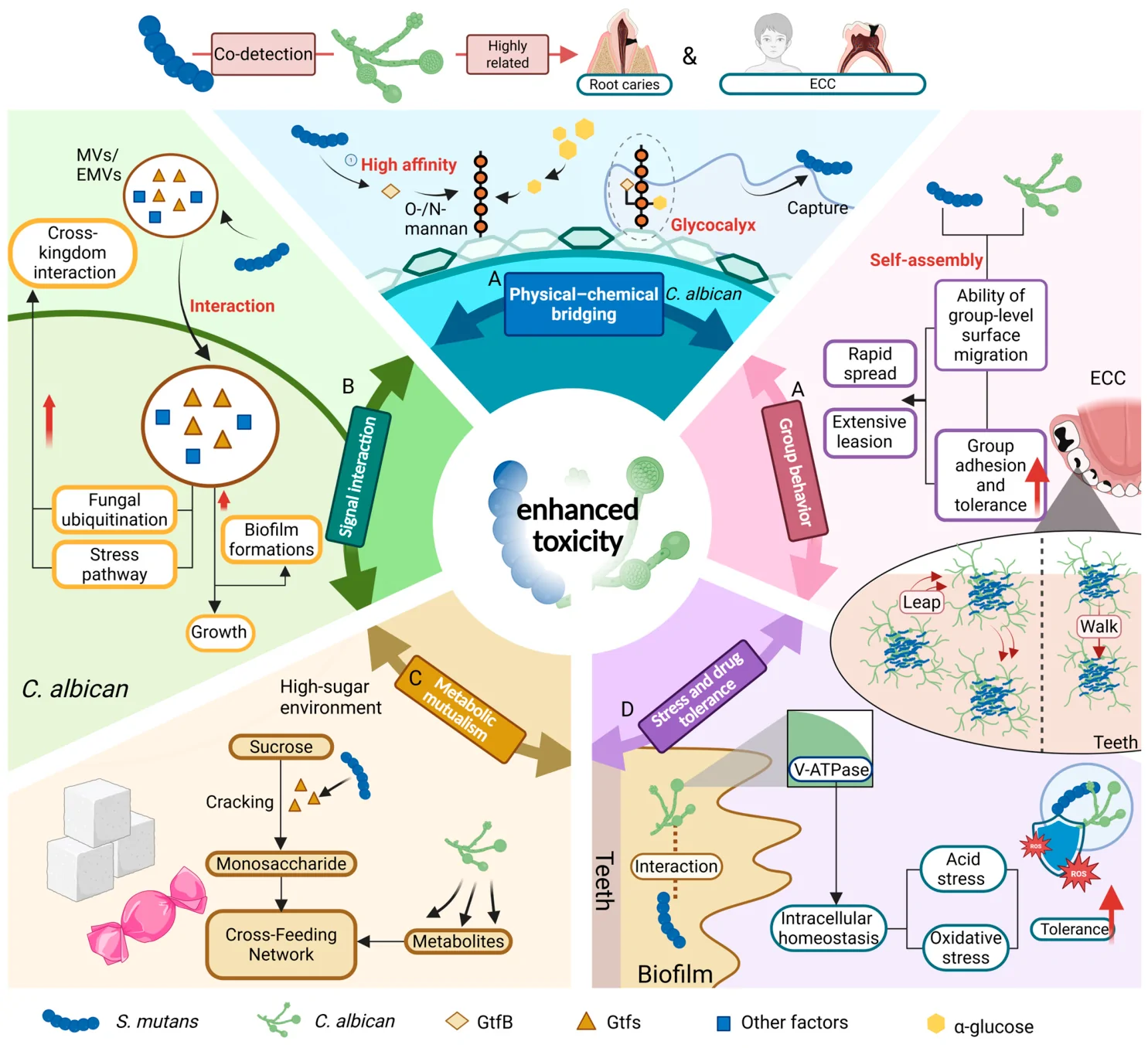
Synergistic interaction between *S. mutans* and *C. albicans* enhances cariogenic virulence. (**A**) The physical–chemical bridging between *S. mutans* GtfB and O-/N-mannan of *C. albicans* facilitates self-assembly into a “superorganism” capable of surface migration via walking or leaping along fungal hyphae; (**B**) active communicating via *S. mutans* MVs/EMVs carrying Gtfs enhances *C. albicans* growth and biofilm formation, and regulates acid/oxidative stress pathways, amplifying cross-kingdom interactions; (**C**) in a high-glucose environment, Gtfs released by *S. mutans* in symbiotic biofilms crack sucrose into monosaccharides, which contribute to a cross-feeding network with fungal metabolites; and (**D**) enhanced tolerance to acid and oxidative stress is mediated by V-ATPase-dependent fungal intracellular homeostasis. MVs/EMVs: membrane vesicles/extracellular membrane vesicles, ECC: early childhood caries. Created in BioRender. J, Bao. (2026) https://BioRender.com/f2rh6jk (access on 2 July 2026).

Mechanistically, the intimate physicochemical interplay between *S. mutans*’ GtfB and N-/O-mannans of *C. albicans* underpins this cross-kingdom association [24,31], facilitating spatial organization and collective behavior. This interaction leads to the self-assembly of the two microorganisms into a cohesive “superorganism”, capable of rapid surface migration via fungal hyphae [26]. Beyond physical adhesion, active biochemical communication via *S. mutans*’ membrane vesicles/extracellular membrane vesicles (MVs/EMVs) carrying Gtfs, further stimulates *C. albicans* growth and biofilm formation. These MVs/EMVs modulate fungal ubiquitination and stress pathways, amplifying cross-kingdom interactions [32,33]. Additionally, these symbiotic biofilms exhibit augmented nutrient cross-feeding [34] and increased tolerance to acid and oxidation stress, partly through V-ATPase-mediated fungal intracellular homeostasis [35], as shown in Figure 2. Collectively, these observations support the rationale for developing novel therapeutic strategies that specifically disrupt interkingdom biofilm networks while preserving oral microbial homeostasis.

### 3.2. Periodontal Disease

Periodontal diseases, the irreversible chronic inflammatory conditions initiated by dental plaque biofilms, trigger progressive destruction of periodontal support tissues [36,37]. Imbalanced biofilm communities can exacerbate the host’s immune response, impair immune surveillance, and trigger systemic inflammation, which further damages both soft and hard periodontal tissues [38,39]. Globally, severe periodontal disease affects approximately 1 billion people [40]. *Porphyromonas gingivalis*, a key pathogen in the development and progression of periodontal disease, plays a central role in oral biofilm dysbiosis and activation of inflammatory responses via its virulence factors, such as gingipains, which disrupt host immune regulation and promote tissue destruction [19,41]. Echoing observations in caries, fungal–bacteria cross-kingdom interactions in periodontal niches are increasingly recognized, with epidemiological studies frequently reporting co-occurrence of *C. albicans* and major periodontopathogens [42,43]. Fungal adhesin-mediated physical contacts and context-dependent dynamic shifts in partner dominance, particularly under varying oxygen levels, can shelter *P. gingivalis* from oxidative stress, facilitate its persistence within mixed biofilms, and promote immune evasion [44,45]. Furthermore, *P. gingivalis*’ implication in associations with systemic diseases, such as diabetes and Alzheimer’s disease, has been well-established [46,47], reinforcing the importance of effective periodontal disease control.

### 3.3. Mainstream Chemical Adjuncts for Dysbiosis-Related Oral Biofilm Diseases and Their Limitations

In addition to mechanical plaque removal (e.g., brushing and scaling), chemical antimicrobial agents are commonly used as adjuncts to control pathogenic oral biofilms, preventing and treating dental caries and periodontal diseases. Chlorhexidine (CHX), one of the most widely used broad-spectrum antimicrobial agents in dentistry, effectively reduces dental plaques at concentrations of 0.12–0.2% and serves as a useful complement to mechanical cleaning. However, its long-term use is limited by multiple adverse effects [10,48,49,50,51], including discoloration of teeth and tongue, taste alterations, mucosal irritation, antimicrobial tolerance, and, most critically, disrupting oral microbiome homeostasis.

Antibiotics play limited roles in the routine prevention of dental caries, typically reserved for cases of acute pulp–periapical infections. In contrast, antibiotics are often used as adjuncts in moderate to severe and aggressive periodontitis. Nevertheless, widespread misuse of antibiotics has raised growing concerns about antibiotic resistance [52,53]. Clinical studies demonstrate that periodontal pathogens exhibit significant resistance to commonly used antibiotics. For instance, *P. gingivalis* shows reduced sensitivity to azithromycin and metronidazole, while *Aggregatibacter actinomycetemcomitans* has developed resistance mutations to tetracyclines [11,54]. These limitations highlight the urgent need for alternative therapeutic strategies that are effective yet less disruptive to the oral ecosystem.

## 4. Pharmacological Basis for CBD’s Antibiofilm, Antivirulence, and Host Response Effects

**Figure 3:**
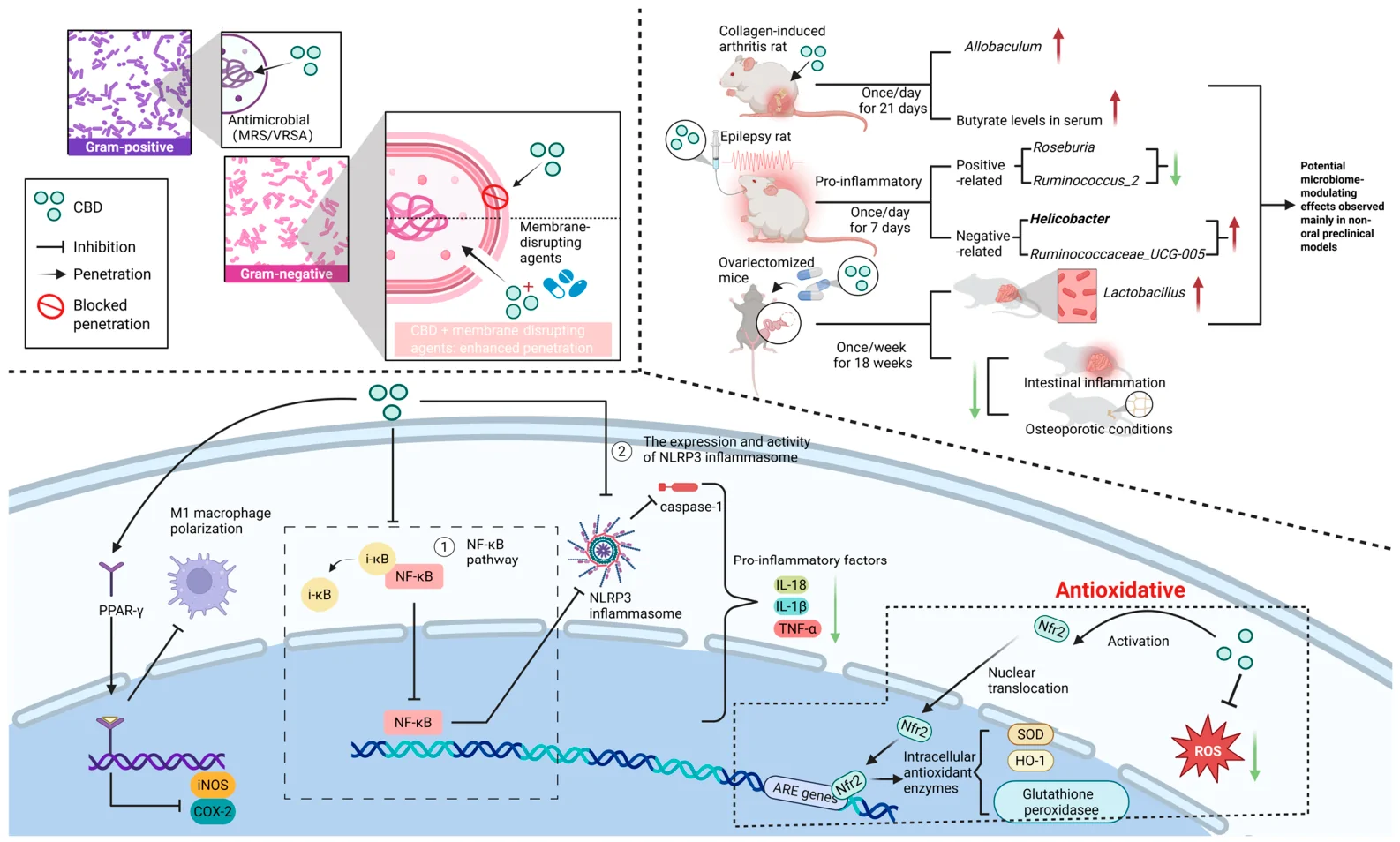
Pharmacological properties of CBD. CBD significantly inhibits Gram-positive bacteria (MRSA, VRSA) and, when used with membrane disruptors such as polymyxin B, overcomes the deficiency of limited activity against Gram-negative bacteria like *Pseudomonas aeruginosa*. Furthermore, it regulates microbiota homeostasis in disease models. CBD activates Nrf2/ARE to upregulate SOD, HO-1, GPx, and NQO1, reduce ROS and alleviate oxidative stress and inflammation, reduce the levels of NLRP3, caspase-1, *IL-1β*, *IL-18*, and *TNF-α* by inhibiting the NF-κB pathway, and suppress iNOS/COX-2 via PPAR-γ. PPAR-γ: peroxisome proliferator-activated receptor gamma, iNOS: inducible nitric oxide synthase, COX-2: cyclooxygenase-2, SOD: superoxide dismutase, HO-1: heme oxygenase-1, ROS: reactive oxygen species. Created in BioRender. J, Bao. (2026) https://BioRender.com/5xw946p (access on 2 July 2026).

The medicinal use of cannabis plant extracts dates back several centuries [55,56]. Cannabidiol (CBD), a phytocannabinoid derived from the *Cannabis sativa* plant, with a molecular weight of 314 Da, contains a catechol ring (a bisphenolic aromatic ring) substituted with a pentyl group and an alkyl-substituted cyclohexene terpene ring [57,58,59]. Unlike tetrahydrocannabinol (THC), CBD is non-psychoactive, is better accepted clinically, and is relatively abundant, ranging from 5% to 10% in different cannabis strains [58,60]. A growing body of preclinical and clinical work indicates CBD’s broad pharmacological spectrum, including microbiome regulation and antimicrobial, anti-inflammatory, antioxidant, analgesic, anticancer, antiepileptic, and neuroprotective effects (Figure 3). While its clinical applications are still in the early stages, its potential is evident. In 2018, the U.S. FDA approved the first CBD-based formulation, Epidiolex^®^, for the treatment of refractory epilepsy (Dravet syndrome and Lennox–Gastaut syndrome), followed by approval by the European Medicines Agency (EMA) for oral treatment of epilepsy in 2019 [61]. CBD’s natural origin, unique structure, and multi-target pharmacological activities provide a strong rationale for exploring its potential in complex, microbiome- and inflammation-driven conditions, including dental caries and periodontal diseases.

### 4.1. Basic Pharmacology and Safety of CBD

CBD exerts its diverse effects through a combination of endocannabinoid-dependent and -independent mechanisms. Despite its low affinity for CB1 and CB2 receptors, CBD indirectly regulates the endogenous cannabinoid system, particularly via the CB2 receptor. This interaction is crucial to CBD’s anti-inflammatory and immune-modulatory effects, whereas the precise mechanisms remain under active investigation and some aspects are still debated [55,62].

Regarding safety, the World Health Organization (WHO) has classified CBD as a substance with “overall good tolerance and high safety” [63]. The CBD-related hepatic risks appear to be dose-dependent in vivo. No cases of drug-induced liver damage have been reported for daily doses below 300 mg of CBD [64], whereas a recent randomized clinical trial, employing moderate doses (~400 mg/day; 5 mg/kg/day for 28 days), observed transient elevations in liver enzymes in 5.6% (8/141) of participants, which normalized within 1 to 2 weeks after discontinuation [65]. Human abuse potential studies show that subjective measures, like drug liking and the likelihood of taking the drug again, remain within placebo ranges even at therapeutic and supratherapeutic doses [61]. Overall, CBD’s adverse effects are generally mild and dose-related, including increased hepatic enzymes, xerostomia, diarrhea, somnolence, and gastrointestinal discomfort. No significant public health risks have been identified thus far.

### 4.2. Antimicrobial and Microbial Homeostasis Rebalancing Activity

CBD exhibits a distinct microbiology-focused profile relevant to oral biofilm-associated diseases, with three themes: (i) potent direct antimicrobial and antibiofilm activity, particularly against Gram-positive bacteria; (ii) synergy with conventional antibiotics, including against resistant strains; and (iii) an emerging capacity to modulate microbial community composition and rebalance dysbiotic gut microbiota.

#### 4.2.1. Potent Antimicrobial Efficacy

In vitro studies consistently indicate that CBD has potent antimicrobial activity against various Gram-positive pathogens, including methicillin-resistant *Staphylococcus aureus* (MRSA) and vancomycin-resistant *S. aureus* (VRSA), as well as antibiofilm formation properties [66,67,68,69]. The minimum inhibitory concentration (MIC) of CBD for these strains typically ranges from 0.5 to 4 μg/mL, with some strains exhibiting MIC values as low as 0.5 μg/mL [67]. In contrast, Gram-negative pathogens exhibit higher tolerance to CBD due to the protective outer membrane barrier. However, this barrier can be pharmacologically bypassed. When combined with membrane-disrupting agents, such as polymyxin B, CBD becomes effective against drug-resistant *Pseudomonas aeruginosa* and other Gram-negative pathogens.

While the mechanism requires further elucidation, converging data indicate that CBD’s antibacterial effect is predominantly membrane-directed. In *S. aureus*, CBD induces rapid depolarization of the cytoplasmic membrane and loss of permeability control, accompanied by a near-simultaneous shutdown of DNA, RNA, protein, phospholipid and peptidoglycan synthesis at bactericidal concentrations, with half-maximal effects occurring at a CBD level close to the MIC. This pattern is consistent with a primary collapse of membrane function rather than selective blockade of a single biosynthetic target. Membrane potential measurements, together with bacterial cytological profiling, further reveal a rapid, concentration-dependent dissipation of the proton motive force and uptake of normally impermeant nucleic acid dyes in *S. aureus* and *Bacillus subtilis*, indicating acute disruption of cytoplasmic membrane integrity [57,69]. Preliminary work also suggests CBD may act as a quorum-sensing (QS) inhibitor, by impairing the universal autoinducer-2 (AI-2) and interfering with AI-2 receptor signaling, thereby suppressing QS-controlled traits such as motility, biofilm formation and virulence-associated gene expression [70,71]. In line with observations for other phytocannabinoids, such as cannabigerol, LuxO activity is increased and the master QS regulator LuxR in *V. harveyi* is repressed [72].

#### 4.2.2. Synergistic Effects with Other Antibiotics

A second key pattern is the synergistic interaction of CBD with established antibiotics, including enhancing bacterial susceptibility and expanding antibiotics’ effective spectrum [73,74]. For example, CBD increases the sensitivity of *S. aureus* to kanamycin and inhibits the release of MVs from Gram-negative pathogens, thereby enhancing the effectiveness of several antibiotics, including rifampin, vancomycin, and erythromycin, against *Escherichia coli* VCS257 [73]. The synergistic effect remains effective against a variety of resistant bacteria, including MRSA and methicillin-resistant *Staphylococcus epidermidis* (MRSE) [74]. Importantly, the available data suggest that the propensity for bacteria to develop resistance to CBD is relatively low. In MRSA, the MIC increased by only ~1.5-fold after repeated exposure, a much slower shift than typically observed for conventional antibiotics [57]. Together, CBD may function as an adjuvant that both potentiates antibiotic efficacy, including against resistant strains, and may help delay resistance development.

#### 4.2.3. Rebalancing Gut Microbiota Homeostasis

Beyond direct antimicrobial effects, emerging studies reveal that CBD can modulate the microbial community structure and help restore dysbiotic microbiota, particularly in the gut. In collagen-induced arthritis rats, 21-day CBD administration at 17.5 mg/kg or 35 mg/kg partially reversed gut microbiota dysbiosis, increased the abundance of short-chain fatty acid-producing genera, such as *Allobaculum*, and elevated serum butyrate levels [75]. In an epilepsy rat model, CBD treatment for 7 days at 20 and 100 mg/kg similarly mitigated gut microbiota dysbiosis [76]. Furthermore, in ovariectomized mice, oral CBD (25 mg/kg) given for 18 weeks increased the relative abundance of *Lactobacillus* in the gut and alleviated intestinal inflammation and osteoporotic conditions [77]. Across these models, CBD tends to enrich beneficial, metabolically supportive taxa, like short-chain fatty acid producers, while reducing inflammatory or dysbiosis-associated profiles, in parallel with improvements in systemic inflammatory or metabolic outcomes. However, current evidence is limited to a small number of preclinical studies in rodent gut models, and the underlying mechanisms are not yet clearly defined to explore whether they are mediated primarily through direct microbial effects, immune modulation, or both. These findings provide indirect biological plausibility for microbiome modulation but should not be extrapolated to the oral cavity.

### 4.3. Anti-Inflammatory, Immunoregulatory, and Antioxidant Effects

With broad molecular targets, including endogenous cannabinoid receptors, the transient receptor potential vanilloid 1 (TRPV1), serotonin 1A (5-HT1A) receptors, and adenosine A receptors, CBD exhibits potent anti-inflammatory and immunomodulatory functions [55]. Studies have demonstrated that CBD inhibits the degradation of inhibitor of κB (IκB), preventing the nuclear translocation of nuclear factor κB (NF-κB) and the transcription of pro-inflammatory genes [78]. Additionally, CBD interferes with the formation and activation of the NLRP3 inflammasome through multiple mechanisms, including inhibition of the NF-κB pathway, which prevents inflammasome activation [79], and directly reducing the expression and activity of NLRP3 and downstream components, such as caspase-1, and interleukins (IL-1β/IL-18) [79,80]. Furthermore, CBD activates peroxisome proliferator-activated receptor gamma (PPAR-γ), a nuclear receptor transcription factor whose activation inhibits the expression of inducible nitric oxide synthase (iNOS) and cyclooxygenase-2 (COX-2), both pro-inflammatory enzymes, and suppresses M1 macrophage polarization [78]. In summary, CBD achieves networked regulation of inflammation and immune responses through its multi-target properties.

CBD exerting dual antioxidant actions could act as a direct free radical scavenger via its hydroxyl group, and indirectly induce cellular antioxidant defense mechanisms. Studies show that CBD upregulates the nuclear factor erythroid 2-related factor 2/antioxidant response element (Nrf2/ARE) pathway, increasing the expression of intracellular antioxidant enzymes such as superoxide dismutase (SOD), glutathione peroxidase, and heme oxygenase-1 (HO-1), while reducing reactive oxygen species (ROS) levels [81,82,83]. In a 5-fluorouracil-induced oral mucositis in vitro and in vivo model, CBD alleviated excessive ROS production, enhanced antioxidant capacity, and reduced inflammation by increasing Nrf2 expression and upregulating the expression of antioxidants such as HO-1 and NQO1 [84].

## 5. CBD’s Implications in Dysbiosis-Related Oral Biofilm Diseases

### 5.1. In Vitro Antibacterial and Antibiofilm Activities of Cbd Against Cariogenic Microorganisms

Across existing in vitro studies, CBD, its biosynthetic precursor cannabigerol (CBG) and the endocannabinoid, Anandamide (AEA), consistently display activity against cariogenic *S. mutans* and *C. albicans* in monoculture settings [85,86,87,88,89,90,91,92], as summarized in Table 1.

MIC, minimum inhibitory concentration. MBIC, minimum biofilm inhibitory concentration. MIC values against *S. mutans* typically fall within the low range (~5 μg/mL for CBD and 2–10 μg/mL for CBG), and most studies report concomitant reductions in viable counts, biofilm biomass and extracellular polysaccharide (EPS) production, indicating a robust anti-cariogenic signal at the mono-species level. Beyond simple growth inhibition, several lines of evidence indicate that cannabinoids can interfere with both biofilm development and the persistence of established biofilms. Sub-MIC CBD in combination with triclosan further suppresses *S. mutans* activity and biofilm formation compared with either agent alone [86], suggesting the potential for synergistic or additive interactions with existing oral care actives. Notably, CBD’s anti-metabolic effect on preformed *S. mutans* biofilms persisted after the withdrawal of CBD, with minimal regrowth observed up to 48 h post-treatment at 20 μg/mL [85]. Taken together, these findings suggest that cannabinoids can destabilize cariogenic biofilms rather than merely delaying planktonic growth.

Intriguingly, the profile of CBD against *C. albicans* differs from its activity against *S. mutans*. Rather than acting primarily as a fungicidal agent, CBD appears to modulate virulence-associated traits [100,101]. It prevents the yeast-to-hyphal transition, inhibits mono-species biofilm formation, and reduces EPS production. This toxicity regulation phenotype is conceptually attractive for dysbiosis-oriented caries management, as it targets pathogenic behavior rather than simply reducing fungal load. However, the experimental evidence remains limited to a small number of in vitro studies, and the stability of these effects under more complex ecological conditions has yet to be established.

Mechanistic data, though still preliminary, suggest that CBD and CBG may inhibit *S. mutans* through increasing membrane permeability, inducing membrane depolarization, and interfering with QS. Notably, *S. mutans* strains deficient in the comCDE QS system show increased sensitivity to CBG [91], supporting a QS-linked mode of action. Comparative in vitro studies further indicate that CBD- and CBG-containing formulations can achieve zones of inhibition and reductions in colony counts of mixed supragingival plaque that are broadly comparable to 0.2% CHX and superior to several commercial toothpastes [93,94]. However, these assays are largely qualitative and conducted under heterogeneous experimental conditions, limiting direct benchmarking of potency or prediction of clinical non-inferiority.

In essence, cannabinoids, especially CBD, exhibit measurable in vitro antibacterial, antibiofilm and virulence-modulatory effects against cariogenic microorganisms. At the same time, the current evidence base is constrained by several important limitations, including employing the mono-species laboratory-adapted strains; no animals or clinical studies to date have specifically tested CBD for preventing dental caries.

### 5.2. Effects of CBD on Periodontal Diseases

#### 5.2.1. Antimicrobial Effects on Periodontal Pathogens

Several studies have tested CBD against key periodontal pathogens (Table 1). CBD exhibits in vitro activity against *P. gingivalis* (*MI*Cs 1.5 t-5 μg/mL) [95,96], and against both planktonic growth and biofilm formation of *A. actinomycetemcomitans*, with an MIC of 1.56 μg/mL [97]. By contrast, *Treponema denticola* is comparatively tolerant (MIC > 10 μg/mL), and CBD exposure predominantly induces a stress-associated transcriptomic response, including upregulation of resistance-related genes such as *TDE_0895* and *TDE_136* [98]. This species-dependent susceptibility suggests that CBD may remodel periodontal biofilms in a non-uniform manner, potentially suppressing some keystone pathogens while exerting sub-inhibitory, stress-inducing pressure on others. These differential effects raise questions about the long-term ecological and resistance consequences of CBD-based interventions, particularly under conditions where sub-MIC exposures are likely.

#### 5.2.2. In Vitro and Vivo Anti-Inflammatory and Host Response Modulation of CBD

##### In Vitro Evidence

**Figure 4:**
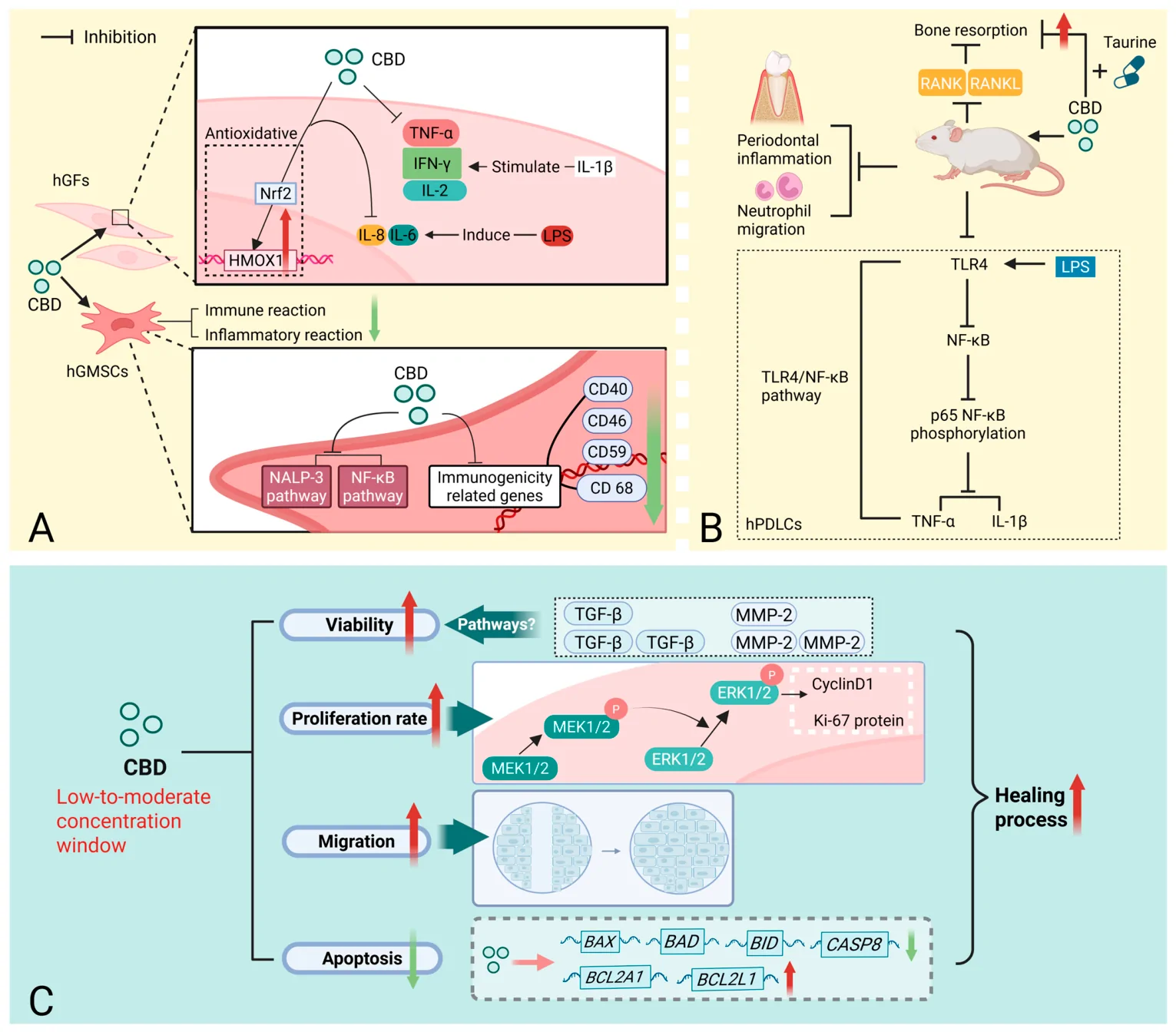
Molecular mechanisms of CBD in regulating periodontal inflammation, immune response and bone remodeling. (**A**) CBD activates Nrf2/HMOX1, reduces TNF-α, IFN-γ, and IL-2 in hGFs, inhibits NALP-3/NF-κB, and downregulates *CD40*, *CD46*, *CD59*, and *CD68* in hGMSCs. (**B**) In periodontitis, CBD inhibits TLR4/NF-κB, reduces p-p65, TNF-α, and IL-1β, and suppresses RANK/RANKL, thereby attenuating neutrophil migration and bone resorption (enhanced by taurine), and promoting healing. (**C**) CBD, exerting biphasic effects on periodontal healing, modulates TGF-β, MMP-2 and cell viability, upregulates Ki-67 and CyclinD1 via MEK1/2-ERK1/2, promotes survival by downregulating *BAX*, *BAD*, *BID*, and *CASP8* and upregulating *BCL2A1* and *BCL2L1* at low doses, whereas high doses induce apoptosis, cycle block, and the upregulation of p53 and *BAX*. RANK: receptor activator of nuclear factor κB. Created in BioRender. J, Bao. (2026) https://BioRender.com/hyif129 (access on 2 July 2026).

Periodontal tissues express cannabinoid receptors, providing a mechanistic basis for the direct host-modulatory actions of CBD. Human gingival fibroblasts (hGFs) express the endogenous CB2 receptor [95], whereas human periodontal ligament cells (hPDLCs) express both CB1 and CB2 receptors, with a shift from CB1-dominated profiles in health toward increased CB2 expression under bacterial inflammatory conditions [102]. Against this receptor background, in vitro studies suggest that CBD possesses anti-inflammatory, immunomodulatory, proliferative and antioxidative properties in periodontal cell types, but in a dose-, time- and cell type-dependent manner (Figure 4).

In hGFs, low-micromolar CBD dampens pro-inflammatory signaling while enhancing cytoprotective pathways. CBD at 0.5 μM produced a modest reduction in LPS-induced IL-6 and IL-8 release, and increased *HMOX1* expression, indicating activation of the Nrf2 pathway, whereas this antioxidant response is lost when cells are primed with LPS [95]. In IL-1β-stimulated hGFs, 1 μg/mL CBD reduces IFN-γ, TNF-α, and IL-2 levels, but does not consistently decrease IL-8, PGE2, IL-6 or IL-12, pointing to an immunoregulatory rather than uniformly anti-inflammatory profile [103]. In contrast, in human periodontal ligament fibroblasts (hPLFs), CBD at 10–20 μM preferentially induces IFN-γ despite preserving cell viability [87], whereas the authors interpreted that increased IFN-γ consequence may be immunosuppression. In human gingival mesenchymal stem cells (hGMSCs), CBD (5 μM) downregulates NLRP3 inflammasome components and NF-κB signaling and skews gene expression toward anti-apoptotic and low-immunogenic phenotypes, mainly via CB1 signaling [104]. In hPDLCs, CBD (2–8 μM) dampens the TLR4/NF-κB activation and reduces IL-1β and TNF-α expression [105].

Taken together, these findings support viewing CBD as a context-dependent immunomodulator rather than a simple anti-inflammatory agent. The apparent therapeutic window is narrow and heterogeneous across cell types: hGFs already show reduced viability above ~1.5 μM, whereas hPLFs tolerate short-term exposure at 10–20 μM. Systematic dose–response and time course studies, including careful control of the CBD–LPS exposure sequence and inclusion of additional clinically relevant cell types such as hPDLSCs, are needed before robust dosing recommendations can be formulated.

##### In Vivo Evidence

Preclinical in vivo studies in rodent periodontitis models generally support a host-protective effect of CBD, although the evidence base remains limited. In ligature-induced periodontitis, topical or systemic CBD at 5 mg/kg/day reduced alveolar bone loss, neutrophil infiltration and gingival expression of TLR4, TNF-α and IL-1β in gingival tissue [105,106,107]. Mechanistically, CBD appears to attenuate bone resorption by suppressing the expression of receptor activator of nuclear factor κB (RANK) and its ligand RANKL in alveolar bone [107]. More recently, combinations of CBD with taurine have been reported to further enhance anti-inflammatory and bone-protective effects in similar models [106], suggesting the potential for adjunctive or synergistic host-modulatory strategies.

#### 5.2.3. CBD’s Biphasic Dose–Response Effect on Pro-Healing Effects

Across periodontal cell models, CBD’s pro-healing actions follow a characteristic biphasic dose–response profile. Within a low-to-moderate concentration window, CBD tends to enhance cell viability, proliferation, migration and matrix production, whereas at higher concentrations, it impairs viability and promotes apoptosis (Figure 4). For example, CBD increased the fibronectin level at 0.025–30 µM, exhibiting a biphasic effect on TGF-β and MMP-2 levels, which increased at lower CBD concentrations and decreased at higher levels [108]. In hGFs, CBD at low concentrations (0.1–1 µg/mL) enhanced hGFs’ metabolic activity, whereas a higher dose (>2 µg/mL) inhibited viability [103]. A separate study in hGFs reported a different effective window, with enhanced metabolic activity at 0.01–10 µM CBD and a reduction at 30 µM, accompanied by increased expression of the proliferation markers cyclin D1 and Ki-67 via activation of the MEK1/2–ERK1/2 pathway [109]. In hGMSCs and hPDLSCs, a low CBD dose favored survival by downregulating pro-apoptotic genes (e.g., *BAD*, *BAX*, *BID*, *CASP8*) [104,110], and upregulating anti-apoptotic genes, including *BCL2A1* and *BCL2L1* [110], whereas high doses of CBD (50 µM) induced increased apoptosis, cycle block, and upregulation of *p53* and *BAX* genes [111].

#### 5.2.4. Clinical Evidence of CBD Against Periodontal Conditions

Clinical evidence remains limited but provides preliminary support for the short-term feasibility of topical CBD formulations. Jirasek et al. conducted a 56-day, placebo-controlled, double-blind randomized trial involving 90 participants with periodontitis. Participants received placebo dental gel plus placebo toothpaste, 1% (*w*/*w*) CBD gel plus 1% CBD toothpaste, or 1% chlorhexidine digluconate gel plus placebo toothpaste. After 56 days, the CBD regimen produced statistically significant improvements in the gingival index, gingival bleeding index, and modified gingival index relative to placebo [95]. However, the CBD and CHX groups received non-equivalent exposure regimens, and the study was not designed to test superiority or non-inferiority. Accordingly, these findings do not establish that CBD is equivalent or superior to CHX. No CBD-related adverse effects were reported during the short follow-up period.

A further clinical study reported improvement in bleeding on probing after the use of a mouthwash containing 1.6% CBD and spilanthol [112]. However, because this was a combination formulation, the independent contribution of CBD cannot be determined. Collectively, the available clinical evidence supports short-term feasibility and hypothesis generation rather than established comparative efficacy.

### 5.3. Additional Supportive Evidence from Other Oral Biofilm-Associated Conditions

Although endodontic infections are not the primary dysbiosis-related focus of this review, they provide supportive evidence for CBD’s activity against resilient oral biofilm-associated pathogens. Endodontic infections are frequently dominated by facultative anaerobes and Gram-positive bacteria, such as *Enterococcus faecalis*, which form robust biofilms within the canal system and potent resilience, contributing to persistent infections [113,114]. Encouragingly, studies have demonstrated that *E. faecalis* is sensitive to CBD, with MICs of 1–8 μg/mL [74,115,116], and CBD can act as a helper compound, decreasing the effective concentration of the antibiotic, bacitracin, against *E. faecalis*. In addition, CBD possessed potent osteogenic differentiation, promoting efficacy in human dental pulp cells (hDPCs) [117,118,119,120]. At a low dose (1.25 µg/mL), CBD recovered the proliferation of LPS-induced human dental pulp stem cells (hDPSCs) and restored their odonto/osteogenic differentiation abilities though Notch activation [117]. CBD treatment also enhanced the osteogenic differentiation of hDPSCs-derived organoid-like microspheroids [118].

### 5.4. Critical Appraisal and Evidence Maturity

Overall, the current evidence base remains preliminary and highly context-dependent. Existing oral studies are largely limited to isolated microorganisms or simplified biofilms, and findings from rodent gut models cannot be directly extrapolated to the oral microbiome. A recent observational study identified different salivary microbial profiles in cannabis users and non-users with periodontitis and found that several of these patterns corresponded to CBD sensitivity or resistance in subsequent planktonic assays [99]. However, the human exposure involved cannabis rather than purified CBD, and the findings therefore cannot establish a causal effect of CBD on the oral microbiome. Moreover, the coexistence of CBD-sensitive and CBD-resistant oral taxa raises the possibility of selective ecological pressure rather than restoration of eubiosis.

The mechanisms underlying CBD-mediated biofilm inhibition remain incompletely defined. It is not yet clear whether CBD primarily reduces microbial load, selectively attenuates cariogenic traits, like acid production, EPS synthesis and stress tolerance, or acts through a combination of these mechanisms. From an ecological perspective, this distinction is important because indiscriminate killing may create ecological vacancies, favor the overgrowth of opportunistic taxa and trigger secondary dysbiosis, whereas selective antivirulence activity may be more compatible with the preservation of microbial homeostasis.

Similar caution is required when interpreting periodontal evidence. Inhibition of isolated periodontal pathogens or fungal virulence does not necessarily predict a net benefit in polymicrobial communities, where microbial interactions are highly dependent on environmental conditions, such as oxygen tension and heme [121], and can be antagonistic. For instance, AI-2 produced by *A. actinomycetemcomitans* suppresses *C. albicans* growth and hyphal formation [122].

The host response evidence is likewise context-dependent, and the effective dose window and timing of CBD administration in periodontal tissues are poorly defined. CBD appears to exert biphasic effects on periodontal cells, with similar patterns observed in other cell types, such as chondrocytes [123]. It is unclear whether the concentrations that are optimal in vitro are achievable and safe at the target sites in vivo. Moreover, the sequence and timing of CBD exposure relative to microbial or inflammatory stimuli (such as LPS) appear to influence the outcomes, with some studies reporting greater efficacy when CBD is applied early in the inflammatory phase [124]. CBD should therefore not be regarded as a uniformly anti-inflammatory or pro-regenerative agent, because its effects vary according to cell type, formulation, concentration, exposure duration, and administration schedule.

Taken together, the current evidence supports biological plausibility rather than established clinical effectiveness. Domain-specific methodological limitations, interpretive boundaries, and qualitative evidence maturity judgments are summarized in Table 2.

### 5.5. Comparative Positioning of CBD Among Established and Emerging Oral Biofilm Adjuncts

The clinical positioning of CBD should be evaluated against agents with different indications and levels of evidence maturity, rather than on the basis of isolated MIC values or inhibition zone assays.

As discussed in Section 3.3, chlorhexidine (CHX) is an established clinical benchmark for adjunctive plaque control, whereas evidence for CBD remains predominantly preclinical. CHX, essential oil (EO) mouthrinses, and cetylpyridinium chloride (CPC) have substantially more mature clinical evidence for plaque and gingivitis control than CBD [125,126,127,128], which has not demonstrated comparable clinical efficacy. Although efficacy remains the primary basis for comparison, potential ecological effects also warrant consideration. For CHX specifically, short-term human studies indicate alterations in salivary microbial community composition [48,129].

Silver nanoparticle (AgNP)-based interventions also remain largely preclinical, with efficacy and toxicity strongly influenced by particle size, coating, silver-ion release, and formulation [130,131]. Unlike CBD, nanosilver fluoride has formulation-specific clinical evidence supporting caries arrest. Nevertheless, these effects cannot be attributed independently to AgNPs because fluoride forms an integral active component [132]. No direct comparison between CBD and AgNP- or nanosilver fluoride-based interventions has been reported.

Probiotics are conceptually more ecology-oriented and may provide modest, strain-specific benefits as adjuncts to non-surgical periodontal therapy, particularly for selected *Lactobacillus reuteri* regimens supported by meta-analytic evidence [133]. However, evidence for caries prevention remains inconsistent and of moderate-to-low certainty [134]. Thus, although clinical evidence for probiotics is more extensive than that for CBD, probiotics remain strain- and indication-specific adjuncts, and no direct comparative studies with CBD are available.

CBD may be attractive as an oral care adjunct because of its non-psychoactive profile, high public visibility, and preclinical evidence of antimicrobial and host-modulatory activity. However, the absence of robust comparative clinical evidence precludes positioning CBD alongside established plaque control agents. CBD should therefore be positioned as an investigational adjunct rather than a substitute for established plaque control agents.

## 6. Safety and Translational Limitations of CBD in Dysbiosis-Related Oral Biofilm Diseases

### 6.1. Safety of CBD in Oral Use

The non-cytotoxic levels of CBD differ among oral cell types in vitro. For hGFs, although results may differ across different studies, CBD concentrations up to 30 μM have generally not affected cell viability [108,109,111]. Similarly, cytotoxicity of CBD was not observed in hPLFs at 20 μM [87], or in oral keratinocytes at 25 μM [111]. Notably, the effective concentrations of CBD used in these studies were all well below the reported non-cytotoxic limits. Xerostomia (dry mouth) is commonly described in clinical trials and pharmacovigilance analyses of cannabinoid-based medicines, including CBD [135,136]. From a dental perspective, this is clinically relevant because chronic hyposalivation increases the risk of dental caries, mucosal infections, and impaired denture retention. Oral topical applications may limit systemic exposure, and short-term trials of CBD-containing topical gels or mouthrinses have not identified xerostomia as a major complaint over 6–8 weeks of follow-up [95,112]. However, this adverse effect warrants attention, particularly in patients with pre-existing salivary gland dysfunction, polypharmacy or long-term use of CBD-based oral products. Larger, adequately powered studies with extended follow-up are required to more precisely define the long-term safety profile of CBD in oral medicine, including the incidence and clinical impact of xerostomia.

### 6.2. Limitations of CBD in Oral Topical Use

The clinical application of CBD in oral cavities is primarily limited by its physicochemical properties and the complexities of the oral environment. CBD is a non-polar molecule with very low solubility in water (approximately 0.0126 mg/mL) [137,138]. Through oral administration, its poor solubility and significant first-pass metabolism result in low bioavailability (around 6% in humans), with considerable individual variability [139,140]. In local oral delivery, CBD’s low solubility leads to uneven drug distribution, making it difficult to effectively penetrate the oral biofilms. Furthermore, CBD is sensitive to temperature, light, and oxygen, which can lead to degradation into various by-products [141,142]. However, it is relatively more stable in oil-based phases [141].

### 6.3. Regulatory, Manufacturing, and Quality Control Considerations

The regulatory status of CBD is product-, indication-, and jurisdiction-specific. In the United States, Epidiolex^®^ remains the only FDA-approved drug containing purified CBD, with indications limited to specific seizure disorders. Classification as hemp-derived does not exempt other CBD products from FDA requirements [143]. No CBD-containing toothpaste, mouthrinse, or gel has been approved for oral biofilm diseases. Epidyolex^®^ holds marketing authorization in the European Union for selected epilepsy-related indications, but no approved indication covers oral diseases [144].

Safety assessments of CBD in foods or cosmetics do not constitute therapeutic approval or evidence of clinical efficacy. For example, the European Food Safety Authority’s provisional intake level applies only to highly purified, non-nanoparticulate CBD supplements and excludes several vulnerable populations, whereas the Scientific Committee on Consumer Safety’s assessment concerns defined CBD and THC concentrations in cosmetic products [145,146]. Regulatory classification and legal availability may therefore differ across countries according to product composition, intended use, and marketing claims.

Clinical translation further depends on pharmaceutical-grade manufacturing rather than consumer-grade hemp products. Manufacturers of therapeutic CBD oral formulations should follow applicable FDA current Good Manufacturing Practice (GMP) requirements or EU GMP standards, with validated control of CBD identity, potency, purity, residual THC, contaminants, stability, raw material traceability, and batch-to-batch consistency [147,148]. Nanocarrier-based systems require additional control of particle size, drug-loading capacity, release profiles, and storage stability [149,150,151]. These measures may help ensure that variation in raw materials, formulation, and manufacturing processes does not compromise product safety, efficacy, or reproducibility.

## 7. Future Directions for CBD in Dysbiosis-Related Oral Biofilm Diseases

CBD exhibits antibiofilm, anti-inflammatory and analgesic activities, and can act against several key cariogenic and periodontopathogenic microorganisms, while modulating host immune responses within the oral cavity. However, widespread consumer use has outpaced rigorous clinical evaluation, standardized quality control, and clear regulatory oversight [152,153,154]. Based on the methodological, safety, formulation, and regulatory limitations outlined in Section 5.4, Section 5.5, Section 6.1, Section 6.2 and Section 6.3, future research should prioritize microbial ecological validation, definition of host-specific therapeutic windows, and development of clinically relevant local delivery systems.

### 7.1. Microbial Ecology and CBD–Biofilm Interactions

Future microbiological research should progress from cross-kingdom models, particularly *S. mutans*–*C. albicans* biofilms, to saliva-derived polymicrobial communities, and animal and clinical studies incorporating microbiome-level analyses. These studies should distinguish direct microbial killing from selective modulation of virulence, fitness, acidogenicity, EPS production, stress tolerance, and cross-kingdom interactions. Microbiota composition and function should be assessed to determine whether CBD can promote ecological recovery or create niches for intrinsically tolerant or opportunistic organisms. Standardized pharmaceutical-grade CBD, clinically achievable exposure regimens, vehicle controls, and established oral care comparators will be necessary for interpretable comparisons.

### 7.2. Host–CBD Interactions and Immuno-Inflammatory Modulation

Future host response studies should define tissue-specific dose–response and time–response relationships. Particular attention should be given to determine how the timing of CBD exposure relative to microbial stimulation affects preventive and therapeutic outcomes. Downstream pathways connecting CB1/CB2 engagement to specific transcriptional networks, pro-resolving mediators and regenerative outcomes have not been systematically mapped, and work on human periodontal ligament stem cells from a regenerative perspective remains scarce. Clarifying these mechanisms is essential for rationally positioning CBD within host-modulatory and regenerative periodontal strategies rather than treating it as a generic anti-inflammatory agent. Long-term, sex-balanced animal studies with active standard-of-care comparators are required to distinguish beneficial immunomodulation and tissue protection from cytotoxicity or excessive immunosuppressive.

### 7.3. Local Delivery Systems and Nanostructured Lipid Carriers

Successful translation of CBD for oral biofilm management will require local delivery systems that improve aqueous dispersion and stability while maintaining sufficient residence time in the oral cavity. Nanostructured lipid carriers (NLCs) may improve CBD dispersion, encapsulation, and release characteristics [151]. Evidence from dermal lipid nanoparticle systems suggests that NLC encapsulation may enhance CBD photostability, attenuate CBD-associated cytotoxicity, and preserve selected anti-inflammatory activities [149,150]. However, these findings have not yet been confirmed on tooth surfaces, within oral biofilms, or in periodontal pockets.

Future oral formulations should prioritize retention under salivary dilution and mechanical clearance, adhesion to relevant oral substrates, and penetration into mature biofilms. Direct oral validation should address local safety, carrier-specific microbiome effects, and reproducible scale-up under GMP-compliant conditions.

## 8. Conclusions

Accumulating evidence supports further investigation of CBD in dysbiosis-related oral biofilm diseases, particularly dental caries and periodontal diseases. The reported effects can be grouped into three interrelated types of activity: antibiofilm activity against oral microorganisms, antivirulence modulation of pathogenic traits such as EPS production, acidogenicity, quorum-sensing-related behavior, and fungal hyphal transition, and host response regulation involving inflammatory, oxidative, immune, bone remodeling, and pro-healing pathways. Nevertheless, the current evidence consists predominantly of in vitro mono-species and other simplified models, while microbiome-level, animal, and clinical data remain limited. Future studies should determine whether CBD can restore or maintain oral microbial homeostasis rather than merely suppress selected pathogens, define safe and effective local dose windows, and develop delivery systems capable of stable retention and controlled release in the complex oral environment.

## Figures and Tables

**Table 1:** The antimicrobial and antibiofilm efficacy of cannabinoids against oral pathogenic microorganisms.

Diseases	Type of Cannabinoids	Model Type	Targeted Pathogen	Origin of Targeted Pathogen	MIC	MBIC	Antibiofilm Effects	Other Results	Toxicity		Reference
									Cell	Concentration	
Caries-related	CBD	Mono-species planktonic & biofilm	*S. mutans UA159*	Laboratory strain	5 µg/mL	5 µg/mL	CBD inhibited *S. mutans* planktonic growth and biofilm formation in a dose-dependent manner, with similar MIC and MBIC values. 5 µg/mL caused decrease in total biomass and metabolic activity in biofilm formation by 90 ± 6% as compared to control. 3 CBD has a strong inhibition on the preformed biofilms regarding the metabolic activity as 10 µg/mL CBD reduced the metabolic activity by 50%. Notably, precoating of the culture plate surfaces with CBD prior to incubation with bacteria inhibited biofilm development. CBD decreases EPS production in *S. mutans* biofilms	At concentrations ≥2.5 µg/mL, CBD prevented the bacteria-mediated reduction in pH values (pH > 6.5). CBD at concentrations of 20 µg/mL and above caused an irreversible anti-metabolic effect even 48 h after drug removal.	/	/	[85]
	CBD	Mono-species planktonic & biofilm	*S. mutans UA159*	Laboratory strain	5 µg/mL	5 µg/mL	A combination of sub-MIC concentrations of CBD and triclosan resulted in reduced viability of the bacteria and prevented biofilm formation, including biofilm metabolic ability and biomass.	The effective concentrations of this drug combination were not cytotoxic.	Vero epithelial cells	CBD was not cytotoxic <25 μg/mL	[86]
	CBD/CBG	Mono-species planktonic	*S. mutans UA159*; *C. albicans ATCC 10231*	Laboratory strain	1. For *S. mutans*, 20 µg for CBD and 10 µg for CBG; 2. For *C. albicans*, >640 µg for both CBD and CBG	/	/	/	Periodontal ligament fibroblasts	CBD and CBG were not cytotoxic ≤20 μM	[87]
	CBG	Mono-species planktonic	*S. mutans UA159*, *S. sanguis 10556*, *S. sobrinus ATCC 27351*, *S. salivarius ATCC 25975*	Laboratory strain	2.5, 1, 5, 5 µg/mL, respectively	/	/	CBG altered *S. mutans* morphology, including elongation and swelling. CBD altered the membrane structures of *S. mutans*, including membrane folding, reduction in the membrane mass and increase in permeability. CBG was able to maintain the pH at 7 for at least 8 h at 2.5 µg/mL and above.	/	/	[88]
	CBG	Mono-species biofilm	*S. mutans UA159*	Laboratory strain	/	/	CBG at 2.5 µg/mL and above reduced the biofilm formation, including biomass, metabolic activity and EPS production. CBG at 2.5 µg/mL and above increased the intracellular reactive ROS Levels; CBG decreased the metabolic activity of preformed biofilms.	2.5 µg/mL CBG reduced the expression of virulence genes related to the cariogenic properties of *S. mutans*, including EPS synthesis genes (*gtfB,C,D*) and quorum sensing genes (*comE*, *comD*, and *luxS*).	/	/	[92]
	CBG	Mono-species biofilm	*S. mutans UA159*	Laboratory strain	/	/	CBG at 2.5 and 5 µg/mL could significantly reduce biofilm toxicity, including decreasing EPS production, disrupting intra-biofilm connectivity, and increasing biofilm porosity.	/	/	/	[89]
	AEA	Mono-species biofilm	*S. mutans UA159*, *S. sobrinus ATCC 27351*, *S. sanguinis ATCC 10556*	Laboratory strain	12.5 µg/mL	12.5 µg/mL	AEA could prevent biofilm formation and cause preformed biofilm reduction. It could reduce EPS secretion. It alters the membrane properties of *S. mutans* and leads to an accumulation of DAPI.	It affects the gene expression of the cell division gene *ftsZ* in *S. mutans*.	Vero epithelial cells	AEA were not cytotoxic ≤50 µg/mL	[90]
	CBD/CBG mouthwash (<1% by weight)	Multi-species planktonic	/	Clinical supergingival plaque	/	/	/	CBD and CBG showed similar zone of inhibition as that of CHX 0.2% The MIC value of CBD and CBG were closer but slightly (2.37 times) higher than that of positive control CHX 0.2%.	/	/	[93]
	CBD/CBG	Multi-species planktonic	/	Clinical supergingival plaque	/	/	/	12.5% CBD and CBG were more effective in reducing the cultivated microorganism colony count in plaques than Oral B, Colgate and Cannabite F toothpastes.	/	/	[94]
	CBD/CBG	Mono-species planktonic	*S. mutans CCM 7409*	Not mentioned	16 µg/mL for CBD; 8 µg/mL for CBG				Human gingival fibroblasts	CBD and CBG were not cytotoxic ≤0.78 and 6.25 µg/mL, respectively	[95]
Periodontal disease-related	CBD/CBG	Multi-species biofilm (subgingival-related)	/	Laboratory strain	/	/	CBD and CBG inhibited the multi-species biofilm formation and reduced the metabolic activity. CBD at 125 µg/mL and CBG at 62 µg/mL reduced biofilm metabolic activity by 50.38% (*p* = 0.03) and 39.9% (*p* = 0.023), respectively.	/	as above	as above	[87]
	CBD/CBG	Mono-species planktonic	*P. gingivalis CCM 3985*	Not mentioned	1.5 µg/mL for CBD, 4 µg/mL for CBG	/	/	/	as above	as above	[95]
	CBD	Mono-species planktonic	*P. gingivalis ATCC 33277*, *Filifactor alocis ATCC 35896*, *T. denticola ATCC 35405*	Laboratory strain	5 µg/mL for *P. gingivalis*, 10 µg/mL for *F. alocis*	*/*	/	CBD at 5–10 μg/mL suppressed the growth of *P. gingivalis* and *F. alocis.* *T. denticola* was resistant to CBD at 0.1–10 μg/mL.	Primary human monocytes	CBD were not cytotoxic ≤5 µg/mL	[96]
	CBD	Mono-species planktonic & biofilm	*Actinomyces naeslundii* (*ATCC 19039*), *Peptostreptococcus anaerobius* (*ATCC 27337*), *Veillonella parvula* (*ATCC 17745*), *Fusobacterium nucleatum* (*ATCC 10953*), *A. actinomycetemcomitans* (*ATCC 43717*)	Laboratory strain	1.56 µg/mL for *V. parvula*, 3.12 µg/mL for *F. nucleatum*, 0.39 µg/mL for *A. naeslundii*, 0.39 µg/mL for *P. anaerobius*, 1.56 µg/mL for *A. actinomycetemcomitans*		CBD at 1.56 µg/mL significantly reduced the biomass of *A. naeslundii*, whereas 3.12 µg/mL resulted in complete killing. CBD at 0.78 µg/mL produced a significant reduction in *A. actinomycetemcomitans* biomass, and at 3.12 µg/mL caused complete bactericidal effect.	/	/	/	[97]
	CBD	Mono-species planktonic	*T. denticola ATCC 35405*	Laboratory strain	>10 µg/mL	/	/	Upon CBD exposure, protective response genes including *TDE_0895*, *TDE_1367* and *TDE_1858*, which possibly related to drug resistance, were upregulated. The downregulated genes exhibited more varied annotations, including functioning in chemotaxis, iron acquisition and proteolysis.	/	/	[98]
	CBD	Human observational salivary microbiome analysis combined with mono-species planktonic susceptibility assays	*A. actinomycetemcomitans*, *F. nucleatum*, *P. gingivalis*, *S. gordonii*, *S. mutans*, *T. forsythia*, *C. gracilis*, *C. durum*, *H. parainfluenzae*, *T. denticola*, *T. putidum* and *V. parvula*	Salivary microbiome from cannabis users and non-users with periodontitis; laboratory strains and low-passage *P. gingivalis* isolates	*A. actinomycetemcomitans*: 1.56 μg/mL; *F. nucleatum*: 1 μg/mL; *P. gingivalis*: 5 μg/mL; *S. gordonii*: 0.1 μg/mL; *S. mutans*: 0.1 μg/mL; *T. forsythia*: 5–10 μg/mL		/	Distinct salivary microbial profiles in cannabis users; multiple *P. gingivalis* strains were CBD-sensitive; ultrastructural abnormalities occurred in CBD-exposed *P. gingivalis*; peptoids enhanced CBD activity against *T. denticola.*			[99]

**Table 2:** Domain-level critical appraisal of the evidence.

Evidence Domain	Critical Appraisal	Interpretive Conclusion	Evidence Maturity
Direct antimicrobial, antibiofilm, and antivirulence evidence [85,86,87,88,89,90,91,92,93,94,95,96,97,98,100,101]	Although multiple phenotypic and mechanistic endpoints were assessed, the evidence remains predominantly derived from in vitro, with heterogeneous cannabinoid formulations, concentrations, comparators, and endpoints. Polymicrobial, host-integrated, and in vivo validation remain limited.	Supports direct activity of CBD and related cannabinoids against selected oral organisms and virulence traits under laboratory conditions, but not broad-spectrum or clinical efficacy.	Preliminary
Periodontal host cell, matrix response, and oral cell safety evidence [95,103,104,108,109,110,111]	Although inflammatory, regenerative, and toxicological outcomes were assessed, the studies involved small numbers of donors, simplified short-term models, and heterogeneous dosing regimens and controls, limiting definition of a clinically relevant local therapeutic window.	CBD effects appear dose-, time-, formulation-, and cell-dependent; regenerative or safety claims remain uncertain.	Preliminary
Experimental periodontitis in animals [105,106,107]	The animal studies were limited by small sample sizes, short durations, male-only rodent models, early treatment initiation, limited dose assessment, and the absence of standard-of-care comparators and microbiome outcomes.	Provides promising preclinical evidence of host protection, but the study designs limit its applicability to chronic human periodontitis.	Preliminary
Clinical periodontal evidence [95,112]	Although the available studies used clinically relevant indices and topical formulations, only two small, short-term studies were identified, and long-term periodontal and microbiome outcomes were not assessed.	Supports short-term feasibility and hypothesis generation, but not routine use or replacement of established plaque control agents.	Limited
Dental pulp and regenerative supportive evidence [117,118,119,120]	Human dental pulp cell studies assessed migration, mineralization, and relevant molecular mechanisms. However, these models provide indirect evidence for oral biofilm diseases, and donor reporting, infected tissue simulation, and in vivo dental validation were limited.	Provides indirect support for regenerative biological plausibility.	Preliminary (indirect)
Oral microbiome rebalancing and ecological safety [75,76,77,99]	No purified CBD intervention study has evaluated restoration of a dysbiotic human oral microbiome, and community-level ecological safety remains undetermined.	Claims that CBD rebalances the oral microbiota are currently unsupported.	Insufficient

Note: Evidence maturity categories represent qualitative narrative judgments and do not constitute a formal GRADE assessment.

## Data Availability

No new data were created or analyzed in this study. Data sharing is not applicable to this article. We thank all the editors and reviewers who participated in the review.
